# Why Do We Need Serological Tests for Severe Acute Respiratory Syndrome Coronavirus-2 Diagnosis?

**DOI:** 10.1089/biores.2020.0026

**Published:** 2020-12-02

**Authors:** Bouchra Ghazi, Adil Elghanmi

**Affiliations:** ^1^ Faculty of Medicine, Mohammed VI University of Health Sciences (UM6SS), Casablanca, Morocco.; ^2^ Faculty of Medicine, Cheikh Khalifa International Hospital, Mohammed VI University of Health Sciences (UM6SS), Casablanca, Morocco.

**Keywords:** diagnosis, humoral response, real-time polymerase chain reaction, SARS-CoV-2, serological tests

## Abstract

Considering the COVID-19 emerging and rapidly evolving situation associated with increased levels of mortality and infectivity risks, the detection and identification of new tests in a fast, safe, and accurate measures would have a high impact regarding prompt clinical and epidemiological management decisions. The combination of real-time polymerase chain reaction and the immunoglobulin class M–immunoglobulin class G antibody serology testing can be a powerful strategy for more accurate severe acute respiratory syndrome coronavirus 2 (SARS-CoV-2) infection diagnosis with less false results slipping through the cracks. The following viewpoint is describing the immunological response to SARS-Cov-2 infection and its implication in the selection of the appropriate diagnosis tools.

Coronaviruses (CoVs) belong to the Orthocoronavirinae's subfamily of the family Coronaviridae. It is a group of enveloped positive-sensed single-stranded RNA viruses. The emerging CoVs have caused recent pandemics of respiratory infectious diseases with high mortality.^1^ In 2003, a novel human coronavirus (severe acute respiratory syndrome coronavirus [SARS-CoV]) was identified as the etiological agent of the SARS-CoV outbreak. In 2011, the world experienced global pandemic caused by Middle East Respiratory Syndrome coronavirus (MERS-CoV).^1^ The latest CoVs to spill over into the human population emerged in Wuhan City, Hubei Province, China, in December 2019. This virus was termed SARS-CoV-2 by the International Committee on Taxonomy of Viruses (ICTV) for SARS-CoV-2. The newly identified virus was responsible for the outbreak in Wuhan and patients with confirmed infection had typical symptoms of fever, cough, shortness of breath, and in severe cases, pneumonia.^2,3^ Worldwide situation spread rapidly, with >34.8 million confirmed cases reported globally and 1 million deaths as of October 4, 2020.^4^ Considering the emerging and rapidly evolving situation associated with increased levels of mortality and infectivity risks, the detection and identification of new tests in a fast, safe, and accurate measures would have a high impact regarding prompt clinical and epidemiological management decisions.^5^

The standard method of suspected infection diagnosis is real-time polymerase chain reaction (PCR), based on viral spike genes, for detection of SARS-CoV-2 nucleic acid in sputum, throat swabs, and secretions of the lower respiratory tract samples.^6,7^ Even though real-time PCR has advantages, this technology has several limitations that warrant some attention in the context of COVID-19.^8–11^ First of all, it incurs a huge cost of instrumentations and consumables, and requires certified laboratories and specifically trained personnel. These tests have limited standardized protocols, and are laborious in operation and time-consuming. Another consideration concerns reported false negatives for real-time PCR of SARS-CoV-2.^12–14^ Increased risk of false negative in pathogen detection, particularly for new emerging or highly variable pathogens is one of major limitations. Any low abundance target will be more likely to experience the Monte Carlo effect because the probability of primer annealing is lower.^15,16^ Because of this, real-time PCR appears weak for accurate data interpretation and is inadequate for rapid and simple diagnosis. A cautionary warning has already been issued for multiple routes transmission and needed strategy to accurately predict infection status.^17^

Current strategy for SARS-CoV-2 diagnosis is to detect viral RNA in oral swabs, which is not perfect and comes with challenges. The virus can be present in anal swabs or blood of patients, whereas oral swabs testing negative.^17^ It has been shown that infected patients can harbor the virus in the intestine at the early or late stage of disease progression. More interestingly, it has been noted that cases with positive blood viremia had negative swabs. Furthermore, the diagnosis of SARS-CoV-2 is confirmed with lower respiratory tract specimens and no upper respiratory tract swabs are investigated for the viral RNA detection, which may conclude bias results during the early infection stage and explain high false negatives in nucleic acid PCR test.^3^ This strongly implies that these patients would likely be considered as SARS-CoV-2 negative through established routine surveillance practices and discharged only on the basis of negative oral swabs, putting them at a high risk of transmission.

For better diagnostic strategy, dissecting the dynamic of immune response to SARS-CoV-2 is essential. Using accumulated knowledge learned from the outbreak of SARS-CoV and MERS-CoV, one can anticipate the gap on human immune responses to SARS-CoV-2 infection.^18^ The neutralizing antibody-mediated humoral response is very potent in neutralizing viral infectivity and prevents reinfection. Small-scale serological screening of SARS-CoV-2 with limited data was reported.^7^ It has been shown that, after the onset of the disease, specific immunoglobulin class M (IgM) reached peak titer in day 9 and immunoglobulin class G (IgG) levels peak within about 2 weeks. Furthermore, patients' serum showed cross-neutralizing activity *in vitro* eliciting a protective humoral immune response. IgG and IgM viral antibodies were identified in all patients regardless of swabs results.^7^ However, it remains to be investigated if kinetic and titer of specific antibody correlates with disease severity. This implied that serological assays can play an important role in SARS-CoV-2 case detection and surveillance. Serology are less likely to miss infected people and can help both indication of immune status and diagnosis of acute infection.

Shedding light on immune responses would be valuable for designing diagnosis and therapeutic intervention for COVID-19. The first contributions to mapping the breadth and kinetics of serological response to SARS-CoV-2 comes from Thevarajan et al.^19^ Authors highlighted that progressive increases in plasma SARS-CoV-2-binding IgM and IgG antibodies happen before the resolution of symptoms and clinical recovery. This immune response to SARS-CoV-2 was described as similar to what we observe in influenza. Using blood samples for diagnosis of SARS-CoV-2 seems to be the best-case scenario for rapid, simple, and highly sensitive detection method. It is well known that IgM antibodies are usually the first to arrive to an infection scene, before high affinity, and longer-lasting IgG responses generation. The detection of IgM antibodies indicates recent exposure to the virus and hence a possible presence in blood. IgM antibodies are often considered as the most sensitive indicator of acute infection. The presence of IgG antibodies generally indicates past exposure and immunity.^20^ Serological screening for both IgM and IgG throughout the time-course of infection could increase diagnostic accuracy, help monitoring course disease during and after treatment, and help identify reservoirs that may amplify outbreaks.^12^ The advantages of serological IgG–IgM combined antibody tests are that they are material and time-saving tools, suitable for testing large samples, require no specialized equipment or technique and can be performed by most hospital or clinic laboratories. Compared with real-time PCR, they are relatively inexpensive, simple to perform, and only requires minimal training.

Gaining a deeper understanding of humoral and cellular immune parameters in larger cohorts of COVID-19 will help predicting disease courses, identifying new tools for diagnosis and disease severity management, and developing protective vaccines with maximum efficacy.^19^ This promising research could also help us identifying earlier patients at risk of severe symptoms and which patients will develop milder cases, based on humoral and cellular immune parameters. It is unclear how the immune system fights SARS-CoV-2 and how it works for recovered patients. Research in this area could provide more insight into how postinfection immunity develops for SARS-CoV-2, and if immunization is temporary or longer-lasting to anticipate seasonal outbreaks.

As asymptomatic carriers could spread SARS-CoV-2 virus and make the current outbreak control more difficult, another promising application of serological tests is screening asymptomatic carriers, which is vital to curb the epidemic.^9,11^ Serological information may simplify and promote contact tracing and surveillance at the local, regional, national, and international levels. Serological immunity can also accelerate return-to-work outcome of frontline health workers, which will be potentially re-exposed to SARS-CoV-2. Antibody testing may be helpful in testing sensitivity of real-time PCR assays for detecting viral infection.

In summary, in the face of a rapidly changing and never-before experienced situation, and unfortunately in the absence of any effective therapeutics or vaccines, we have to leverage the power of testing to overcome the pandemic confronting us. One must be aware that we are facing a new virus and uncharted territory, exposing us to new questions and challenges we had yet to be ready to ponder on: what is the most appropriate test, and for whom and when? What to test? How often to test? And, what to do with test results?

Today, in the thick of the outbreak, the need for a rapid, simple to use, sensitive, and accurate diagnostic test is critical to quickly identify symptomatic and asymptomatic cases of SARS-CoV-2, reduce virus shedding and transmission, and ensure timely treatment of patients. The combination of real-time PCR and the IgM–IgG antibody serology testing can be a powerful strategy for more accurate SARS-CoV-2 infection diagnosis with less false results slipping through the cracks.

## Abbreviations Used

CoVs: coronaviruses
IgG: Immunoglobulin class G
IgM: Immunoglobulin class M
MERS-CoV: Middle East Respiratory Syndrome coronavirus
PCR: polymerase chain reaction
SARS-CoV: severe acute respiratory syndrome coronavirus
